# SC2EGSet: StarCraft II Esport Replay and Game-state Dataset

**DOI:** 10.1038/s41597-023-02510-7

**Published:** 2023-09-08

**Authors:** Andrzej Białecki, Natalia Jakubowska, Paweł Dobrowolski, Piotr Białecki, Leszek Krupiński, Andrzej Szczap, Robert Białecki, Jan Gajewski

**Affiliations:** 1 Warsaw University of Technology, Electronics and Information Technology, Warsaw, Poland; 2 SWPS University, Neurocognitive Research Center, Warsaw, Poland; 3 Polish Academy of Sciences, Institute of Psychology, Warsaw, Poland; 4 Adam Mickiewicz University in Poznań, Mathematics and Computer Science, Poznań, Poland; 5 Józef Piłsudski University of Physical Education in Warsaw, Physical Education, Warsaw, Poland

**Keywords:** Scientific data, Computational science, Databases, Software, Statistics

## Abstract

As a relatively new form of sport, esports offers unparalleled data availability. Our work aims to open esports to a broader scientific community by supplying raw and pre-processed files from StarCraft II esports tournaments. These files can be used in statistical and machine learning modeling tasks and compared to laboratory-based measurements. Additionally, we open-sourced and published all the custom tools that were developed in the process of creating our dataset. These tools include PyTorch and PyTorch Lightning API abstractions to load and model the data. Our dataset contains replays from major and premiere StarCraft II tournaments since 2016. We processed 55 “replaypacks” that contained 17930 files with game-state information. Our dataset is one of the few large publicly available sources of StarCraft II data upon its publication. Analysis of the extracted data holds promise for further Artificial Intelligence (AI), Machine Learning (ML), psychological, Human-Computer Interaction (HCI), and sports-related studies in a variety of supervised and self-supervised tasks.

## Background & Summary

Electronic sports (esports) are a relatively new and exciting multidisciplinary field of study^1,2^. There are multiple groups of stakeholders involved in the business of esports^3^, as well as interest from the academic community.

From the perspective of sports copmpetition, esports is both in its infancy and at the forefront of using analytics to optimize training and performance. New training methods are derived from an ever increasing pool of data and research aimed at generating actionable insights, mostly by applying methods from sport science^4–9^. Rule changes in sports come at varying time intervals and frequently with unpredictable effects on their dynamics. It is especially relevant to share esports data to assess rapid changes in game design and their impact on professional players in the relatively unstructured nature of esports competition and development^10,11^. Regarding academia, esports have been utilized in several fields for diverse purposes. Advancements in Artificial Intelligence (AI) and Machine Learning (ML) have shown that Reinforcement Learning (RL) agents are capable of outmatching human players in many different types of games, including esports^12–15^. Psychological research on neuroplasticity has also shown the potential of esports and video games in general, such as their propensity for inducing structural brain adaptation^16^. Further, previous studies have shown that playing video games can enhance cognitive functioining in a wide range of domains, including perceptual, attentional and spatial ability^17,18^.

As such, esports provide a platform for studying complex task performance in both humans and complex AI systems. Data obtained from esports titles - gathered from high-level tournament performance - may provide a path to improving the quality and reproducibility of research in these fields, owing to the stability of such data relative to that gathered in the wild. A lower technical overhead and greater data availability for modeling could assist further research^19–21^. Despite the digital nature of esports - which are their greatest asset with respect to data gathering - there seems to be a lack of high-quality pre-processed data published for scientific and practical use. The goal of our work is to mitigate this issue by publishing datasets containing StarCraft II replays and pre-processed data from esports events, classified as “Premiere” and “Major” by Liquipedia (https://liquipedia.net/starcraft2/Portal:Leagues) in the timeframe from 2016 until 2022.

StarCraft II is a widely played and longstanding esports title, which can briefly be described as follows:

“StarCraft II: Legacy of The Void (SC2) contains various game modes: 1v1, 2v2, 3v3, 4v4, Archon, and Arcade. The most competitive and esports related mode (1v1) can be classified as a two-person combat, real-time strategy (RTS) game. The goal of the game for each of the competitors is either to destroy all of the opponent’s structures or to make them resign.” Moreover, StarCraft II contains multiple matchmaking options: “Ranked game - Players use a built-in system that selects their opponent based on Matchmaking Rating (MMR) points. Unranked game - Players use a built-in system that selects their opponent based on a hidden MMR - such games do not affect the position in the official ranking. Custom game - Players join the lobby (game room), where all game settings are set and the readiness to play is verified by both players - this mode is used in tournament games. Immediately after the start of the game, players have access to one main structure, which allows for further development and production of units.”^22^.

While reviewing StarCraft II related sources, we were able to find some publicly available datasets made in 2013 “SkillCraft1”^23^ and 2017 “MSC”^24^. These datasets are related to video games and in that regard could be classified as “gaming” datasets. However, it is not clear what percentage of games included within these datasets contain actively competing esports players. Our efforts aim to remedy this while also providing tools to make such data more easily accessible and usable.

A summary of the contributions stemming from this work is as follows: (1) The development of a set of four tools to work with StarCraft II data^25–28^; (2) The publication of a collection of raw replays from various public sources after pre-processing^29^; (3) The processing of raw data and publishing results as a dataset^30^; (4) and the preparation of an official API to interact with our data using PyTorch and PyTorch Lightning for ease of experimentation in further research^31^.

### Related work

In Table 1 we present a comparison of two other StarCraft II datasets to our own. Authors of the SkillCraft1 dataset distinguished the level of players based on the data. They proposed a new feature in the form of the Perception-Action Cycle (PAC), which was calculated from the game data. This research can be viewed as the first step toward developing new training methods and analytical depth in electronic sports. It provided vital information describing different levels of gameplay and optimization in competitive settings^32^.

**Table 1:** StarCraft II dataset comparison.

Dataset	esports	public	replays available	pre-processed	API available	replays	timespan
SC2EGSet^30^	✓	✓	✓	✓	✓	17895	2016–2022
SkillCraft1^23^	✗	✓	✗	✓	✗	3395	ND^+^
MSC^24^	✗	✓	*	✓	✓	36619	ND^+^

*provided by the game publisher.

^+^ND - not disclosed.

There are existing datasets in other games. Due to the major differences in game implementations, these could not be directly compared to our work. Despite that, such publications build upon a similar idea of sharing gaming or esports data for wider scientific audience and should be mentioned. Out of all related work, STARDATA dataset is notable in that it comes from prior generation of StarCraft game. This dataset seems to be the largest StarCraft: Brood War dataset available^33^. Moreover, in the game of League of Legends, a multimodal dataset including physiological data is available^34^.

Related publications focused on in-game player performance analyses and psychological, technical, mechanical or physiological indices. These studies were conducted with use of various video games such as: Overwatch^35,36^, League of Legends^37–41^, Dota 2^42–46^, StarCraft^47–49^, StarCraft II^50–54^, Heroes of the Storm^42^, Rocket League^55^, and Counter-Strike: Global Offensive^56–60^, among others^61^. In some cases a comparison between professional and recreational players was conducted.

Most studies did not provide data as a part of their publication. In other cases, the authors used replays that were provided by the game publishers or were publicly available online - such data collections in their proprietary format are unsuitable for immediate data modeling tasks without prior pre-processing. The researchers used raw files in MPQ (SC2Replay) format with their custom code when dealing with StarCraft II most often built upon existing specifications - s2client-proto^62^. Other studies solved technical problems that are apparent when working with esports data and different sensing technologies, including visualization, but with no publication of data^63–67^. Some researchers have attempted to measure tiredness in an undisclosed game via electroencephalography (EEG)^68^, and player burnout using a multimodal dataset that consisted of EEG, Electromyography (EMG), galvanic skin response (GSR), heart rate (HR), eyetracking, and other physiological measures in esports^69^.

## Methods

### Dataset sources

The files used in the presented information extraction process were publicly available due to a StarCraft 2 community effort. Tournament organizers and tournament administrators for events classified as “Premiere” and “Major” made the replays available immediately after the tournament to share the information with the broader StarCraft II community for research, manual analysis, and in-game improvement. Sources that were searched for replaypacks include Liquipedia, Spawning Tool, Reddit, Twitter, and tournament organizer websites - these are fully specified in the supplemental file. All replaypacks required to construct the dataset were identified and downloaded manually from the publicly available sources. Replaypack sources did not provide any further information on re-distribution. It is customary for tournament administrators to act as judges/referees in case of any issues and have full access to the players PC’s. The mechanism of recording a single replay is automatic and turned on for all of the users that join a game lobby unless turned off on purpose. Recording replays is most often mandatory for tournament gameplay in case of potential disputes between players or technical problems. In case of technical difficulties in tournaments, StarCraft 2 has a built-in mechanism to resume a game from a recorded replay. After the tournament is finished, the administrators with access to all of the replays share them with the StarCraft 2 community for use at will. Ownership of all data before processing with additional software is subject to Blizzard’s EULA and other Blizzard licenses. Therefore, as the tournament organizers shared these data with the public, we deemed no additional permissions required. The entirety of our processing conformed with the “Blizzard StarCraft II AI and Machine Learning License” and Blizzard representatives were contacted before the release of processed data.

### SC2ReSet description

Our raw data release “SC2ReSet: StarCraft II Esport Replaypack Set”^29^ is subject to the original End User License Agreement (EULA) published by Blizzard, and depending on use case can be processed under the “Blizzard StarCraft II AI and Machine Learning License”.

The dataset with raw data contains replaypacks in.zip format, with each compressed archive representing a single tournament. Within each archive all files with the “.SC2Replay” extension are MPQ archives (custom Blizzard format) and hold all information to recreate a game with the game engine or to acquire the game state data with a replay parsing library. Additionally, each archive has a “.json” file with metadata containing a mapping between the current file hash and previous directory structure that may contain information about the tournament stage for a given replay file.

### Dataset pre-processing

**Fig. 1:**
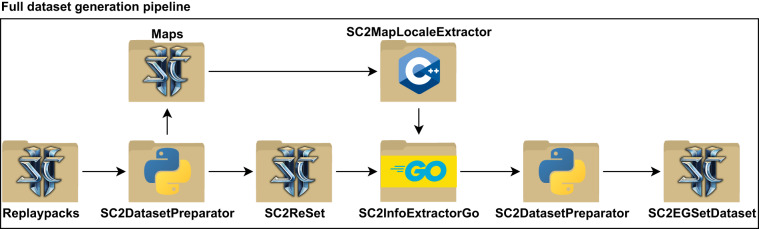
Pre-processing and processing steps of our pipeline that result in SC2ReSet^29^ and SC2EGSetDataset^30^. We used a custom data processing toolset including the SC2DatasetPreparator^26,27^, and SC2InfoExtractorGo^25^.

Dataset pre-processing required the use of a custom toolset. Initially, the Python programming language was used to process the directory structure which held additional tournament stage information. We include this information in the dataset in a separate file for each tournament, effectively mapping the initial directory structure onto the resulting unique hashed filenames. Moreover, a custom tool for downloading the maps was used; only the maps that were used within the replays were downloaded^26^. Finally, to ensure proper translation to English map names in the final data structures, a custom C++ tool implementation was used. Information extraction was performed on map files that contained all necessary localization data^27^. The entirety of our processing pipeline is visualized in Fig. 1.

### Data processing

Custom software was implemented in the Go programming language (Golang) and built upon authorized and public GitHub repositories endorsed by the game publisher - s2prot. The tool was used to perform information extraction from files in MPQ format with the SC2Replay extension. Information extraction was performed for each pre-processed directory that corresponded to a single tournament. Depending on the use case, different processing approaches are possible by providing command line arguments^25^.

### Data parsing and integrity

The parsing capabilities of the tooling were defined with a Golang high-level parser API available on GitHub - s2prot. After initial data-structures were obtained, the next step checked the integrity of the data. This was accomplished by comparing information available across different duplicate data structures that corresponded to: the number of players, map name, length of the player list, game version, and Blizzard map boolean (signifying whether a map was published by Blizzard). If a replay parser or custom integrity check failed, the replay was omitted.

### Data filtering and restructuring

Filtering for different game modes was omitted as collected replay files were a part of esports tournament matches. Most often, StarCraft II tournament matches are played in the form of one versus one player combat. Therefore, it was assumed that filtering for the number of players was not required at this step. Custom data structures were created and populated at this stage. This allowed for more control over the processing, summary generation, and final output. Merging data structures containing duplicate information was performed where applicable.

### Summarization and JSON Output to zip archive

Replay summarization was required in order to provide information that can be accessed without unpacking the dataset. Finally, the data was converted from Golang data structures into JavaScript Object Notation (JSON) format, and compressed into a zip archive.

## Data Records

### Dataset description

The final dataset “SC2EGSet: StarCraft II Esport Game State Dataset”^30^ is indexed on Zenodo and published under the CC BY 4.0 International conforming with the “Blizzard StarCraft II AI and Machine Learning License”. This dataset was processed using tools that were a derivative work of officially endorsed and published replay parsing specification. Blizzard representatives were contacted before the release of processed data to ensure if there are no issues with the dataset publication in respect to potential licensing infringement.

Each tournament in the dataset is a.zip file. Within each archive there are 5 files: (1) Nested.zip with the processed data named “ReplaypackName_data.zip”; (2) Main log of the processing tool used for extraction named “main_log.log”; (3) Secondary log in JSON format containing two fields “processedFiles” and “failedToProcess” that contains a list of files that were parsed, e.g. “processed_failed_0.json”; (4) Mapping between the current file hash and previous directory structure named “processed_mapping.json” as introduced in Section SC2ReSet Description; (5) JSON file with descriptive statistics named “ReplaypackName_summary.json” containing information such as: game version histogram, dates at which the observed matches were played, server information, picked race information, match length, detected spawned units, and race picked versus game time histogram.

### Dataset properties

The collected dataset consisted of 55 tournaments. Within the available tournaments, 18309 matches were processed. The final processing yielded 17895 files. While inspecting the processed data, we observed three major game versions. The critical properties of our work are as follows:

- To secure the availability of the raw replays for further research and extraction with other toolsets built by the community, the SC2ReSet: StarCraft II Esport Replaypack Set was created^29^.
- The replays were processed under the licenses provided by the game publisher: “End User License Agreement (EULA)”, and “Blizzard StarCraft II AI and Machine Learning License”.
- Our dataset is released under CC BY 4.0 International to comply with Blizzard EULA and the aforementioned “Blizzard StarCraft II AI and Machine Learning License”.

### Data fields

The top level fields available in each JSON represent a single StarCraft II replay. All of the available events and fields not listed here are described in the API documentation for the JSON parser in our repository - https://github.com/Kaszanas/SC2_Datasets, which loads the data for further experiments^31^. Interpretation for some of the fields is not included due to the limitations of the official publisher documentation - s2protocol documentation. Additionally, some of the fields acquired from the parsing steps are left as duplicates and can be used to verify the soundness of the provided data.

#### details

Field containing arbitrary “details” on the processed StarCraft II game.

- gameSpeed: Game speed setting as set in the game options. Can be one of “slower”, “slow”, “normal”, “fast”, or “faster”. Typically, competitive or ranked games are played on the “faster” setting. Additional information is available at: https://liquipedia.net/starcraft2/Game_Speed,
- isBlizzardMap: Specifies if the map that was used in replay was approved and published by Blizzard (game publisher),
- timeUTC: Denotes the time at which the game was started.

#### header

Field containing details available in the header of the processed StarCraft II match.

- elapsedGameLoops: Specifies how many game loops (game-engine ticks) the game lasted,
- version: Specifies the game version that players used to play the game.

#### initData

Field containing details on the game initialization.

- GameDescription: contains information such as the GameOptions.

#### metadata

Field that contains some game “metadata”. Available fields are:

- baseBuild: build number of the game engine,
- dataBuild: number of the build,
- gameVersion: game version number,
- mapName: name of the map the game was played on.

#### gameEvents

Field that contains a list of game events with additional information on player actions. For a full description of nested fields in each of the available events please refer to the official API documentation as referenced in the API repository provided along with the dataset - https://github.com/Kaszanas/SC2_Datasets^31^. Available events are:

- CameraSave: user saved the camera location to be accessible by a hotkey,
- CameraUpdate: user updated the camera view,
- ControlGroupUpdate: user updated a control group,
- GameUserLeave: denotes an event that occurs when user left the game,
- UserOptions: denotes the game settings that the user has set,
- Cmd,
- CmdUpdateTargetPoint,
- CommandManagerState,
- SelectionDelta.

#### messageEvents

Field that contains a list of events with additional information on player messages. Available events are:

- Chat: denotes that a player wrote something in the in-game chat.

#### trackerEvents

Field that contains a list of “tracker” events. Available types of events are:

- PlayerStats: information on the economy of the players,
- PlayerSetup: contains basic information mapping userId to playerId to slotId,
- UnitInit: when the player initializes production of a unit,
- UnitBorn: when the unit spawns,
- UnitDied: when the unit stops existing,
- Upgrade: when an upgrade finishes,
- UnitDone,
- UnitOwnerChange,
- UnitPositions,
- UnitTypeChange.

#### ToonPlayerDescMap

Field that contains an object mapping the unique player ID to additional information on the player’s metadata.

## Technical Validation

### Technical validation description

Technical validation for the raw replay files contained in “SC2ReSet: StarCraft II Esport Replaypack Set”^29^ is built into the tools that were used to create the dataset and was introduced in the method section as processing steps. We assume that all of the replays that went through our process of data extraction are correct and up to the Blizzard’s shared specification for replay parsing.

**Fig. 2:**
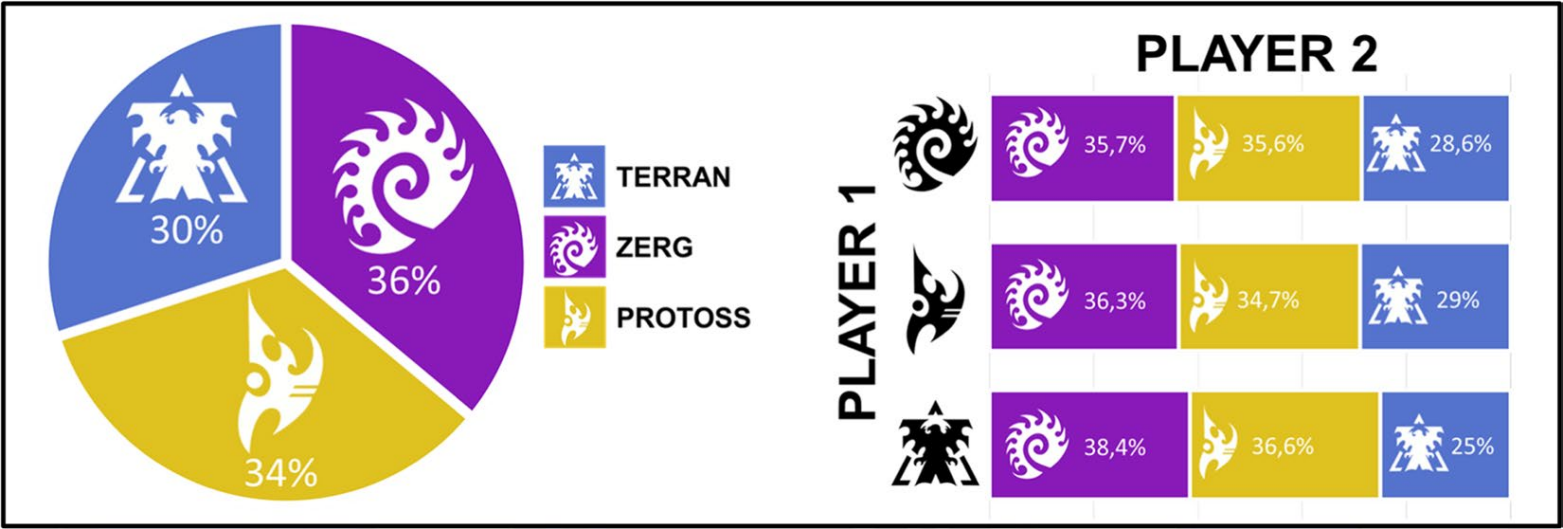
Distribution of player races and race matchup information.

**Fig. 3:**
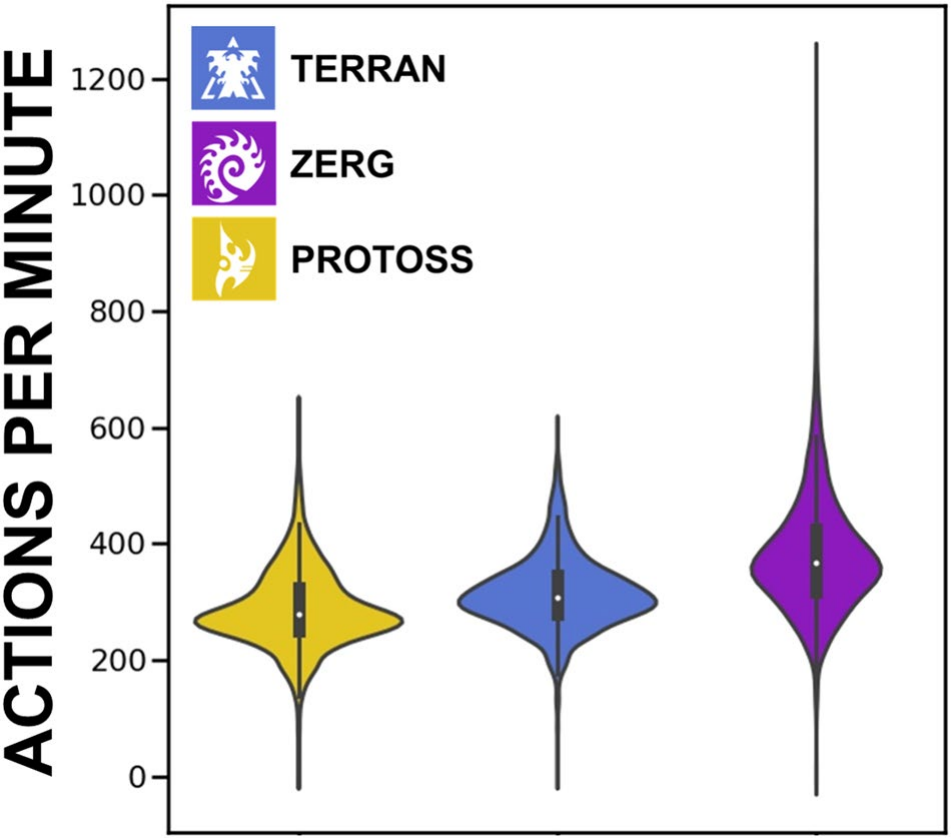
Actions per minute (APM) by player race.

Validating the “SC2EGSet: StarCraft II Esport Game State Dataset”^30^ included manual and visual verification and scripted checks for the soundness of obtained data. Processing logs are attached to each of the manipulated replaypacks. Figure 2 depicts the frequency with which each of the races played against the other and the distribution of races observed within the tournaments. Figure 3 depicts the distribution of match times that were observed.

**Fig. 4:**
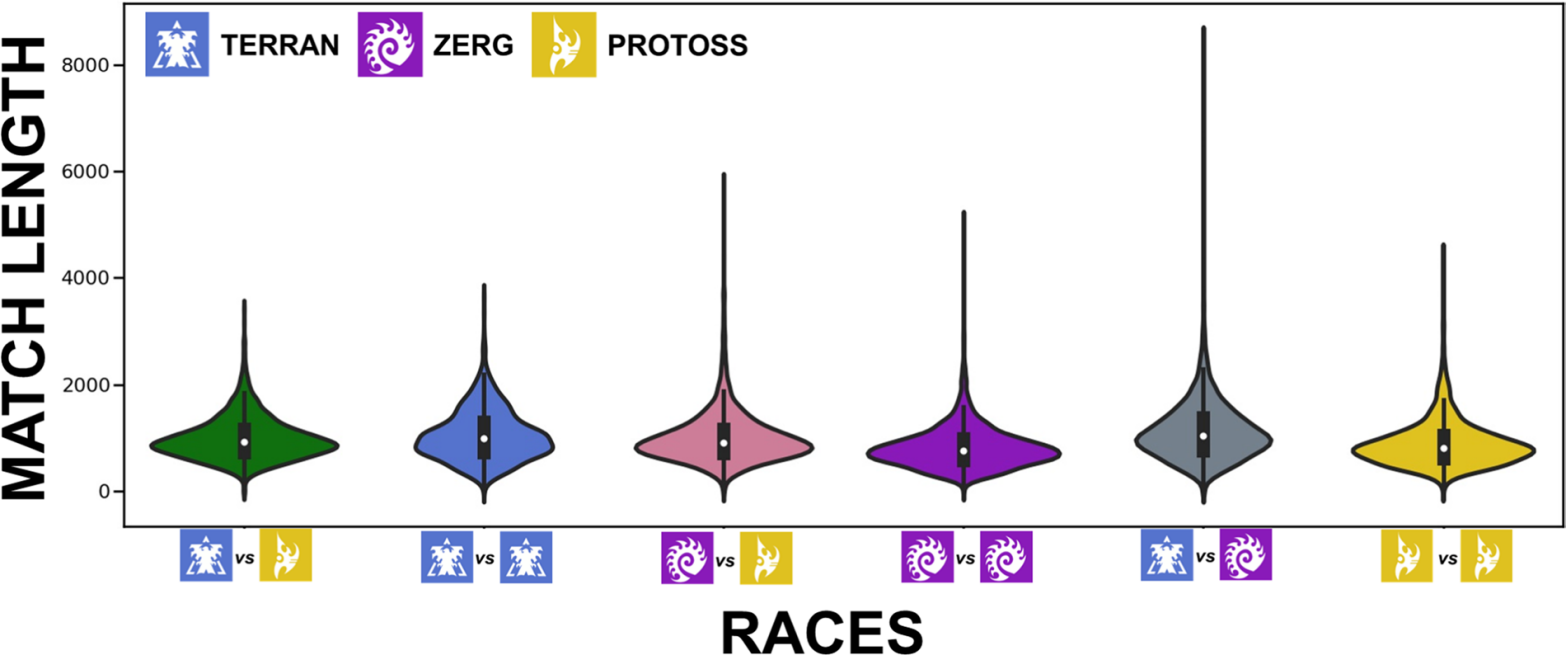
Match time distribution split by races: Terran (blue), Protoss (yellow), and Zerg (purple).

The oldest observed tournament was IEM 10 Taipei, which was played in 2016. The most recent observed tournament was IEM Katowice, which finished on 2022.02.27. The game contains different “races” that differ in the mechanics required for gameplay. Figure 4 shows visible differences in the distribution of match time for players that picked different races.

### Technical validation experiments

To further ensure the technical validity of our data, we performed two sets of classification experiments using different model implementations.

#### Data preparation

Matches were initially filtered to only include those exceeding or equaling a length of 9 minutes, which is approximately the 25th percentile of match length values. Next, a set of features was generated from the available economy-related indicators. Additional features were generated by combining mineral and vespene indicators into summed resource indicators. Match data were then aggregated by averaging across match time for each player, resulting in 22,230 samples of averaged match data (from 11,115 unique matches). Standard deviations were computed in addition to averaged values where applicable. Further features were then generated by computing ratios of resources killed/resources lost for army, economy and technology contexts, along with a ratio of food made to food used. As a final step, prior to feature standardization, each feature was filtered for outliers (replacing with median) that exceeded an upper limit of 3 standard deviations from the feature mean.

#### Feature selection

The feature count was reduced by first computing point biserial correlations between features and match outcome, selecting for features with a statistically significant (*α* = 0.001) coefficient value exceeding that of ±0.050. Next, a matrix of correlations was computed for the remaining features and redundant features were removed. As a result, 17 features remained after this process, of which 8 were basic features (mineralsLostArmy, mineralsKilledArmy, mineralsLostEconomy, mineralsKilledEconomy, and the SD for each).

#### Modelling

Modelling was conducted on features (economic indicators) that represented the global average gamestate, in which all time points were aggregated into a single state, and also as a time series in which the gamestate was represented at a sampling rate of approximately 7 seconds. Three algorithms were chosen for comparative purposes: Logistic Regression (sklearn.linear_model.LogisticRegression), Support Vector Machine (sklearn.svm.SVC)^70,71^, and Extreme Gradient Boosting (xgboost.XGBClassifier)^72^. Each algorithm was initiated with settings aimed at binary classification and with typical starting hyperparameters. A 5-fold cross validation procedure was implemented across the models.

Two sets of models were trained for the average gamestate and one for the gamestate as a time series. In the first averaged set of models the input features represented the economic gamestate of a single player without reference to their opponent, with the model output representing outcome prediction accuracy for that player - a binary classification problem on scalar win/loss classes. The second averaged set of models differed in that it used the averaged economic gamestate of both players as input features, and attempted to predict the outcome of “Player 1” for each match. Finally, the time series models consisted of a feature input vector containing the economic gamestate at 7 second intervals - the task here was also to predict the outcome of a match based on only a single player’s economic features, as in the single-player averaged set of models.

Label counts were equalized to the minimal label count prior to generating the data folds, resulting in 10,744 samples of “Win” and “Loss” labels each for the single-player averaged models and the time series models. For the two-player set of averaged models (containing the features of both players in a given match), the total number of matches used was 10,440. Accuracy was chosen as the model performance evaluation metric in all three cases. Computation was performed on a standard desktop-class PC without additional resources.

#### Results

**Fig. 5:**
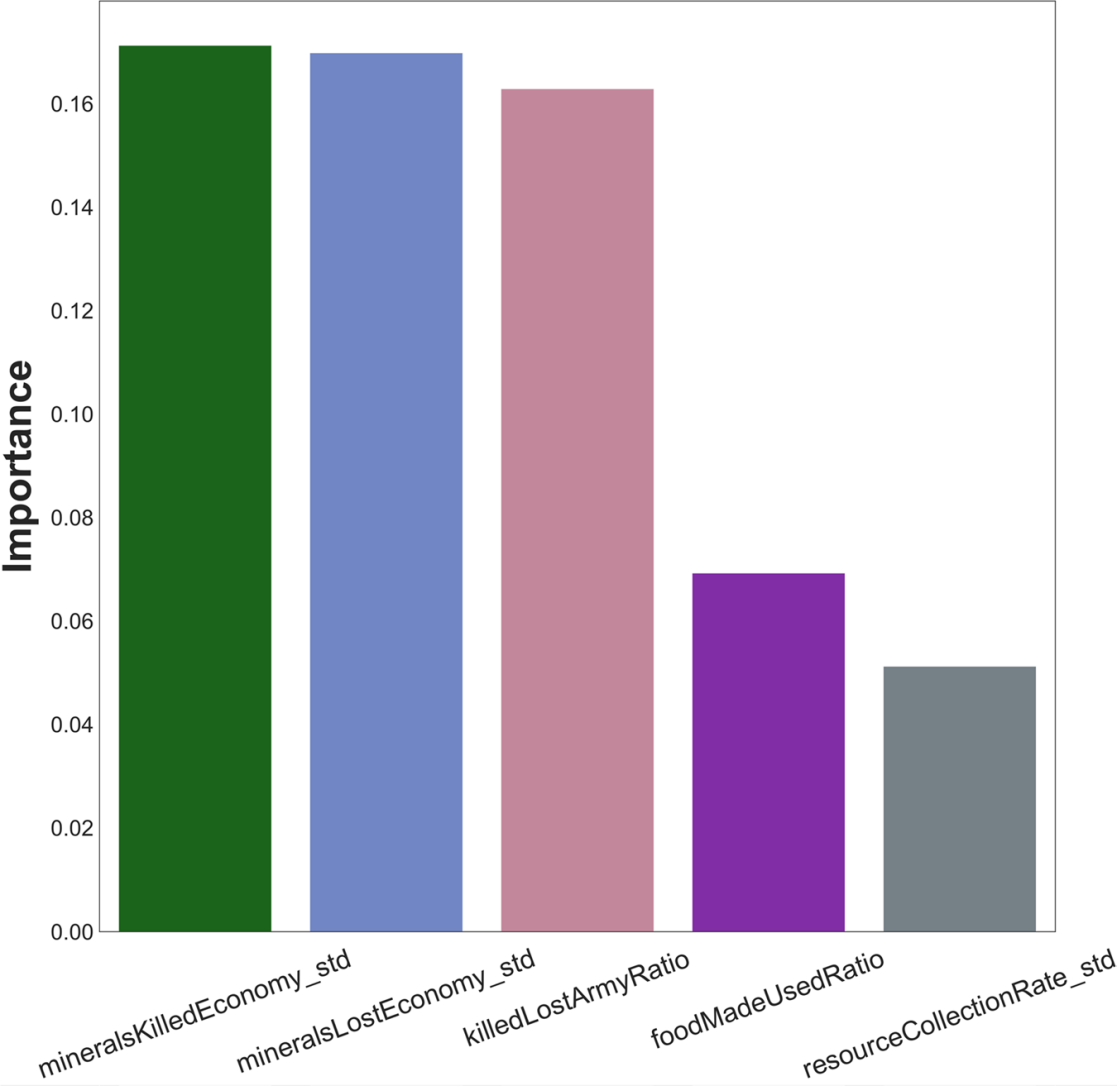
Percentages of feature importances based on XGBoost fit to all data.

As the results indicate (see Table 2), good outcome prediction can be achieved from economic indicators only, even without exhaustive optimization of each model’s hyperparameters. For the one-player averaged set of models, SVM and XGBoost displayed similar performance, with the logistic classifier lagging slightly behind. For the two-player averaged set of models, all three algorithms performed essentially equally well. Feature importances were taken from a single-player XGBoost model (with identical hyperparameters) that was applied to the entire dataset for illustrative purposes. Figure 5 depicts the top five features by importance. It is interesting to note that importance was more heavily centered around mineral-related features than those tied to vespene, which is likely tied to how mineral and vespene needs are distributed across unit/building/technology costs. Further feature investigation is required to verify this tendency.

**Table 2:** Classification models and their performance metrics for two separate win prediction models.

Classifier	Accuracy	SD	Hyperparameters
One Player Prediction			
Support Vector Machine - RBF	0.8488	0.0075	kernel=‘rbf’, C=10, gamma=‘auto’
XGBoost	0.8397	0.0064	Booster=‘gbtree’, eta=0.2, max_depth=5
Logistic Regression	0.8118	0.0057	C=10, penalty=‘l2’
Two Player Prediction			
Support Vector Machine - RBF	0.9071	0.0055	kernel=‘rbf’, C=10, gamma=‘auto’
XGBoost	0.8924	0.0063	Booster=‘gbtree’, eta=0.2, max_depth=5
Logistic Regression	0.8916	0.0063	C=10, penalty=‘l2’

The “One Player Prediction” models attempt to correctly output if one of the players won or lost. The “Two Player Prediction” models have access to the data for both of the players and attempts to output if “Player 1” won or lost.

**Fig. 6:**
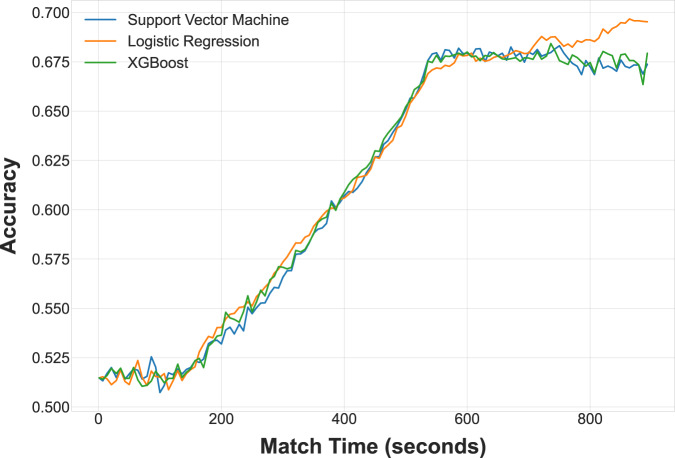
Accuracy comparison of applied classification models.

Figure 6 depicts the time series application of these models as an illustration of outcome prediction accuracy over time. It should be noted that these time series results are not based on any form of data aggregation, and as such only basic economic features could be used for classification (18 features in total).

Each timepoint contains the average accuracy for 5-fold cross validation, with a minimum match length of 9 minutes and a maximum match length of approximately 15 minutes. All three algorithms provided similar performance over time, although this may be an effect of the minimal hyperparameter optimization that was performed. Further, it is also interesting to note and that all three algorithms meet a classification performance asymptote at approximately the same match time (550 seconds), which may indicate that this is where economic indicators begin to lose their predictive power and (presumably) other factors such as army size, composition, and their application become the primary determinants. The code for our experiments is available at a dedicated GitHub repository: https://github.com/Kaszanas/SC2EGSet_article_experiments.

## Usage Notes

### Dataset loading

Interacting with the dataset is possible via PyTorch^73^ and PyTorch Lightning^74^ abstractions. Our API implementations exposes a few key features: (1) Automatic dataset downloading and extraction from Zenodo archive; (2) Custom validators that filter or verify the integrity of the dataset; (3) The ability of our abstractions to load and use any other dataset that was pre-processed using our toolset.

**Fig. 7:**
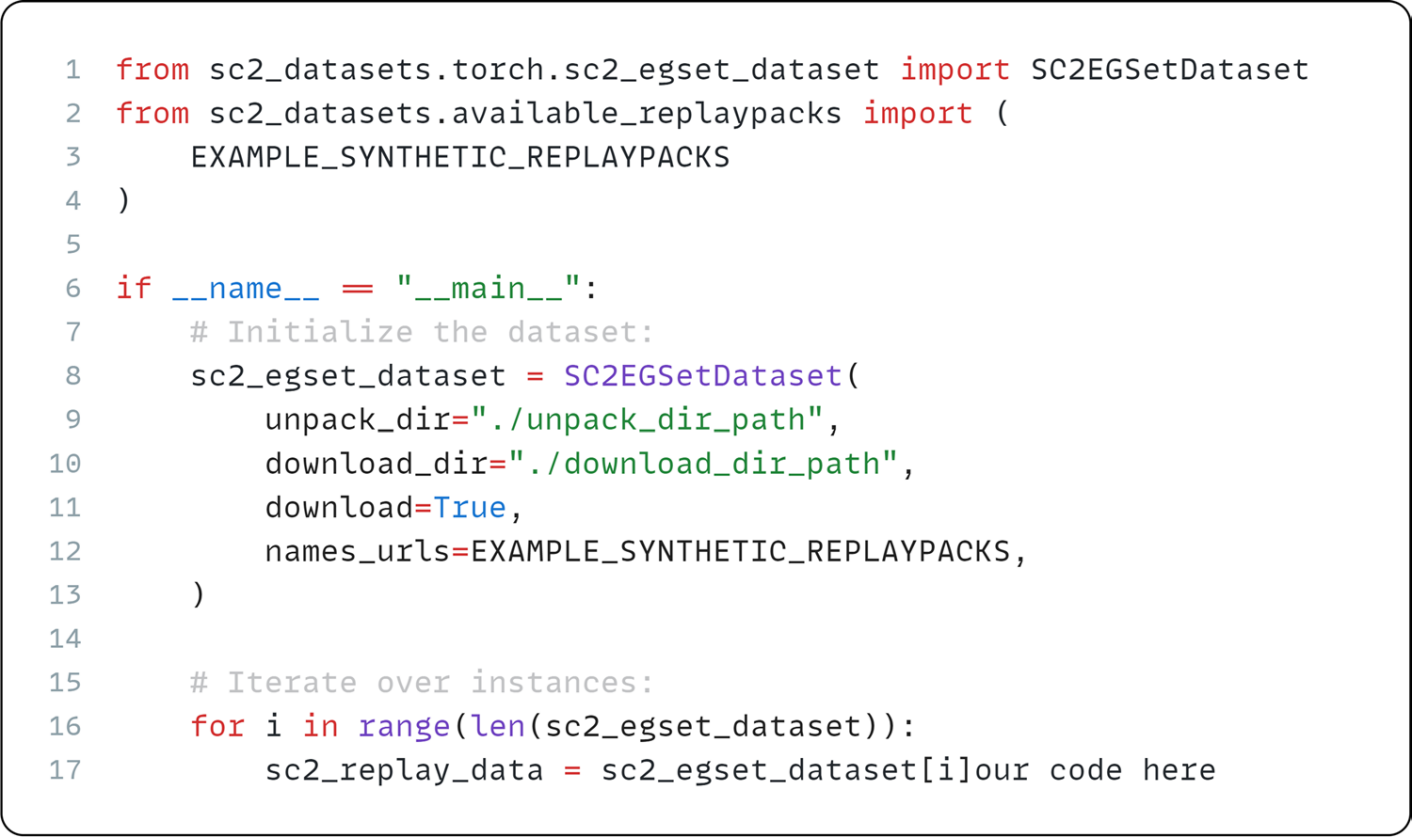
Example use of the SC2EGSetDataset class with PyTorch with a synthetic replaypack prepared for testing.

**Fig. 8:**
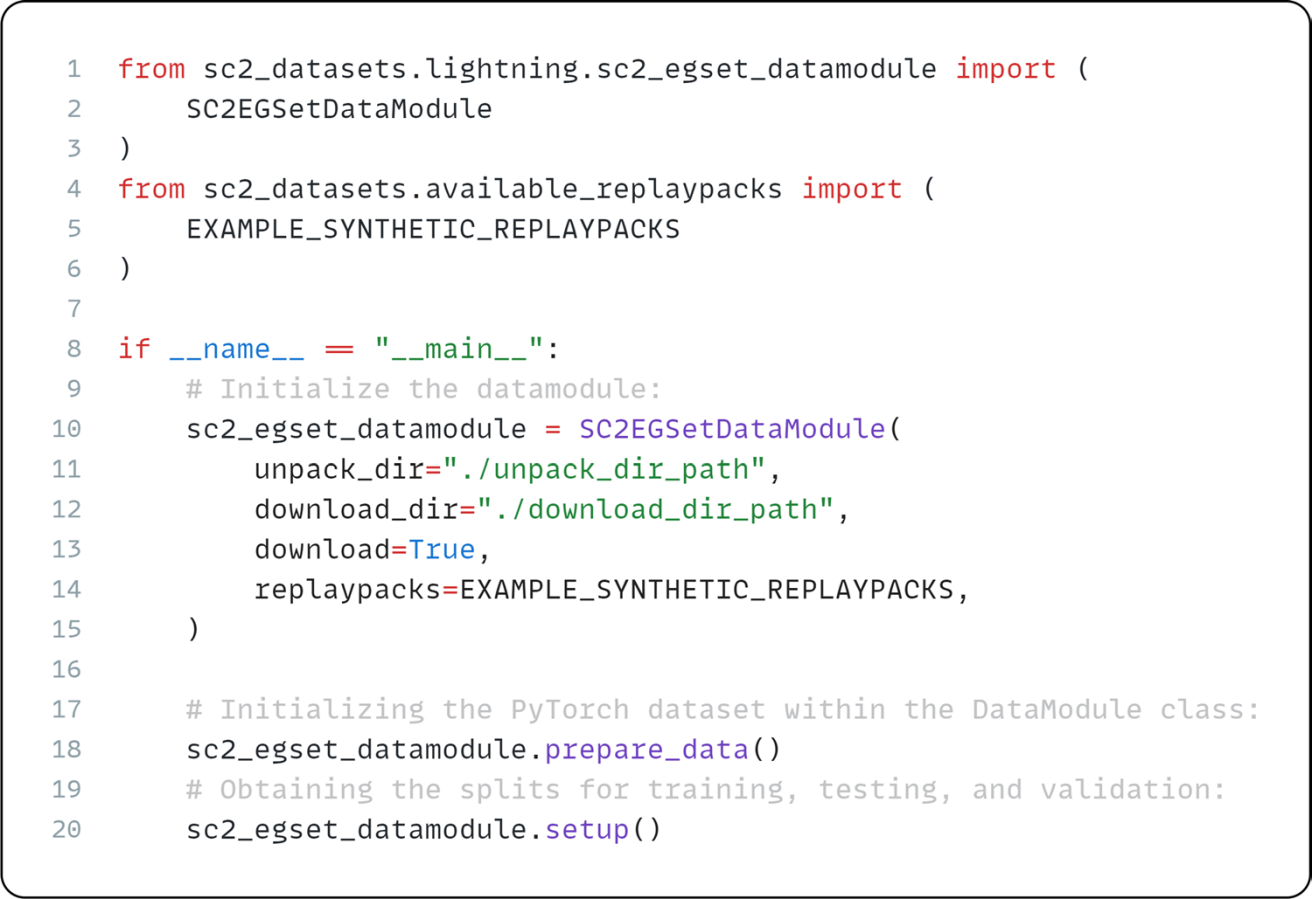
Example use of the SC2EGSetDataModule class with Lightning using a synthetic replaypack prepared for testing.

The required disk space to succesfully download and extract our dataset is approximately 170 gigabytes. We showcase the example use of our API in Fig. 7. Please note that the API^31^ is subject to change and any users should refer to the official documentation as referenced in the API repository for the latest release features and usage information - https://github.com/Kaszanas/SC2_Datasets. Additional listing showcasing the use of a pre-defined SC2EGSetDataset class through our API is shown below in Fig. 8.

### Limitations

We acknowledge that our work is not without limitations. The design and implementation of our dataset do not consider the ability to obtain StarCraft II data through game-engine simulation at a much higher resolution. Because of this, the extracted dataset cannot reflect exact unit positioning. Replays in their original MPQ (SC2Replay) format contain all necessary information to recreate a game using game-engine API. Therefore, we plan to continue our research and provide more datasets that will expand the scientific possibilities within gaming and esports. Further, it should be noted that the experiments described here are more illustrative than investigative in nature, and could be greatly expanded upon in future work. Additionally, due to many changes to the game over time some data fields may be set to have a value of zero; in such cases any further usage of this datasets needs to take into consideration that the representation of a game changed over time. We recommend further research to use SC2ReSet^29^ to compute game-engine simulated information. We do not provide simulation observation data that allows more detailed spatiotemporal information to be extracted at a higher computational cost. Moreover, it is worth noting that the dataset completeness was dependent on which tournament organizers and tournament administrators decided to publish replaypacks.

Additionally, we would like to note that this dataset consists only of the tournament-level esports replays and their data. In that regard there is no possibility to do more generalized analyses that would take into consideration people of varying skill levels.

Future authors may want to filter out replays that ended prematurely due to unknown reasons. Our dataset may contain replays that are undesirable for esports research. We have decided against the deletion of replays to preserve the initial distributions of data. Additionally, as filtering was omitted (besides that performed for the purposes of the described experiments), there is a risk that the dataset contains matches that were a part of the tournament itself but did not count towards the tournament standings. Due to the timeframe of the tournaments and game version changes, despite our best efforts, some information might be missing or corrupted and is subject to further processing and research.

### Supplementary information

SC2EGSet Supplemental File

## Data Availability

Our dataset was created by using open-source tools that were published with separate digital object identifiers (doi) minted for each of the repositories. These tools are indexed on Zenodo^25–27^. We have made available a PyTorch^73^ and PyTorch Lightning^74^ API published to PyPI for accessing our dataset and performing various analyses. Additionally, Our API is accessible in the form of a GitHub repository - https://github.com/Kaszanas/SC2_Datasets, which is available on Zenodo with a separate doi. All of the instructions for accessing the data and specific field documentation are published there^31^. The code used for technical validation experiments is available for preview in a GitHub repository: https://github.com/Kaszanas/SC2EGSet_article_experiments. In the process of preparing this article, PyTorch Lightning has changed its name into Lightning. We have decided to use the old form of the name, following the citation template provided by the Lightning project on GitHub^74^. GitHub Links to the tooling used in the dataset preparation: • https://github.com/Kaszanas/SC2InfoExtractorGo, • https://github.com/Kaszanas/SC2DatasetPreparator, • https://github.com/Kaszanas/SC2MapLocaleExtractor, The official dataset API is available at the following repository: https://github.com/Kaszanas/SC2_Datasets. Additional tooling for potential anonymization tasks with data from private collections is available at: https://github.com/Kaszanas/SC2AnonServerPy.
