# Supplementary material for: SC2EGSet: StarCraft II Esport Replay and Game-state Dataset
Source: Sci Data. 2023 Sep 8;10:600. doi: 10.1038/s41597-023-02510-7 (PMC10491788; doi:10.1038/s41597-023-02510-7)
Supplement: Supplementary file 1 — SC2EGSet Supplemental File [file 41597_2023_2510_MOESM1_ESM.pdf]

1 Data Links

As the initial collection of the data for "SC2ReSet: StarCraft II Esport Replaypack Set" [1] (archived under citation 29 in the main manuscript) was concluded based on publicly existing sources we list the websites from which we have acquired these replaypacks. Some source material is unavailable as the links from SpawningTool or Liquipedia point to websites that no longer exists. Below are the links that point to specific data repositories and private clouds hosting the source data.

- 2016 IEM 10 Taipei
source link:
https://tl.net/forum/starcraft-2/503418-iem-taipei-replaypack,
download link:
http://www.mediafire.com/download/kt9i1ei9i901s1c/IEM_10_Taipei_Replaypack.zip.
- 2016 IEM 11 Shanghai
source link:
https://tl.net/forum/sc2-tournaments/512267-iem-shanghai-replaypack,
download link:
Not available at the time of publication.
- 2016 WCS Winter
source link:
https://liquipedia.net/starcraft2/2016_WCS_Winter_Circuit_Championship
download link:
http://media.blizzard.com/wcs/wcs-winter-2016.zip.
- 2017 HomeStory Cup XV
source link:
https://twitter.com/TrAiDoS/status/889247940530896902,
download link:
https://www.dropbox.com/sh/sh31fg0b6eoc13l/AAAEUOMDmictnT_fnuk4urfHa?dl=0.
- 2017 HomeStory Cup XVI
source link:
https://twitter.com/TrAiDoS/status/929835968768724992,
download link:
https://www.dropbox.com/s/hafx7ex2bnryzow/HomeStory%20Cup%20XVI%20Replay%20Pack.rar?dl=0.
- 2017 IEM Shanghai
source link:
https://liquipedia.net/starcraft2/IEM_Season_XII_-_Shanghai,
download link:
https://www.mediafire.com/file/225blfk2zi1882r/IEM+Shanghai+2017+Replays.zip.
- 2017 IEM XI World Championship Katowice
source link:
https://liquipedia.net/starcraft2/IEM_Season_XI_-_World_Championship,
download link:
https://www.mediafire.com/file/4er2bk8k5d65bb4/IEM+XI+-+World+Championship+-+StarCraft+II+Replays.rar.
- 2017 WCS Austin
source link:
https://news.blizzard.com/en-us/starcraft2/20719815/wcs-austin-2017-replays-available,
download link:
https://bnetcmsus-a.akamaihd.net/cms/kb_media/eg/EGN44QD7A4PY1494021225497.zip.
- 2017 WCS Global Finals
source link:
https://liquipedia.net/starcraft2/2017_WCS_Global_Finals,
download link:
https://bnetcmsus-a.akamaihd.net/cms/content_entry_media/5d/5DR7RFI2XJH91510960969376.zip.
- 2017 WCS Jonkoping
source link:

https://liquipedia.net/starcraft2/2017_WCS_J%C3%B6nk%C3%B6ping,
download link:
https://bnetcmsus-a.akamaihd.net/cms/content_entry_media/yr/YR2VHXLN69FK1498242102371.zip.

- 2017 WCS Montreal
source link:
https://liquipedia.net/starcraft2/2017_WCS_Montreal,
download link:
https://bnetcmsus-a.akamaihd.net/cms/content_entry_media/it/ITCX5YL35EZF1505095577501.zip.
- 2017 WESG Barcelona
source link:
https://lotv.spawningtool.com/replaypacks/,
download link:
https://drive.google.com/open?id=1EY3rFtOgrGsiVdpPX8H7qYlUmxPqwwi_.
- 2017 WESG Haikou
source link:
https://liquipedia.net/starcraft2/World_Electronic_Sports_Games_2017,
download link:
https://drive.google.com/drive/folders/1x2qfNQl47UIgNqhIOTHaAliH0tL69Rlp.
- 2018 Cheeseadelphia 8
source link:
https://twitter.com/Joe_LoGuidice/status/1072661196183080961,
download link:
Not available at the time of publication.
- 2018 HomeStory Cup XVII
source link:
https://twitter.com/TrAiDoS/status/1013583844275081216,
download link:
https://www.dropbox.com/s/7lrneeg1e4y60ut/HomeStory%20Cup%20XVII%20Replay%20Pack.rar?dl=0.
- 2018 HomeStory Cup XVIII
source link:
https://twitter.com/TrAiDoS/status/1066825582774812672,
download link:
https://drive.google.com/drive/folders/1SjbiVSRbKyj4iSMzm8NXtIlmelSkudvX.
- 2018 IEM Katowice
source link:
https://liquipedia.net/starcraft2/IEM_Season_XII_-_World_Championship,
download link:
https://www.dropbox.com/s/0s532uwfiok4q6c/IEM_Katowice_Replays.zip?dl=0.
- 2018 IEM PyeongChang
source link:
https://liquipedia.net/starcraft2/IEM_Season_XII_-_PyeongChang,
download link:
https://www.dropbox.com/s/rpcfixuqt07jk73/IEM%20PyeongChang%20Replays_180207.zip?dl=0.
- 2018 WCS Austin
source link:
https://liquipedia.net/starcraft2/2018_WCS_Austin,
download link:
https://bnetcmsus-a.akamaihd.net/cms/page_media/6s/6S3XIMI7F39H1528210488967.zip.
- 2018 WCS Global Finals
source link:
https://liquipedia.net/starcraft2/2018_WCS_Global_Finals,
download link:
https://bnetcmsus-a.akamaihd.net/cms/gallery/bd/BDSWARJ0UNJ61541302045188.zip.
- 2018 WCS Leipzig
source link:

https://liquipedia.net/starcraft2/2018_WCS_Leipzig,
download link:
https://bnetcmsus-a.akamaihd.net/cms/content_folder_media/i0/I0SUSYYIUE8Y1517362143803.zip.

- 2018 WCS Montreal
source link:
https://liquipedia.net/starcraft2/2018_WCS_Montreal,
download link:
https://bnetcmsus-a.akamaihd.net/cms/page_media/aa/AAM2IIU01GV01536692179246.zip.
- 2018 WCS Valencia
source link:
https://liquipedia.net/starcraft2/2018_WCS_Valencia,
download link:
https://bnetcmsus-a.akamaihd.net/cms/content_entry_media/c3/C39ILK2S0SHG1532034006985.zip.
- 2018 WESG Grand Finals
source link:
https://liquipedia.net/starcraft2/World_Electronic_Sports_Games_2018,
download link:
https://drive.google.com/file/d/1OMLB-Otus1SQ6QgfjFNJ4kNilg9XxFum/view.
- 2019 Assembly Summer
source link:
https://liquipedia.net/starcraft2/Assembly_Summer/2019,
download link:
https://drive.google.com/drive/u/0/folders/18MbLC4b5b-Ain6HorZR9u968K0Cep2hr.
- 2019 HomeStory Cup XIX
source link:
https://twitter.com/TrAiDoS/status/1145481556196282368?s=20,
download link:
https://drive.google.com/drive/folders/19wxjJYI8VGCVxNC8PXvrcuTwwgqyqta4.
- 2019 HomeStory Cup XX
source link:
https://liquipedia.net/starcraft2/HomeStory_Cup/20,
download link:
https://drive.google.com/drive/folders/1EtbWl7QwUMy2vsJWAmWuAvn0orLkvhrr.
- 2019 IEM Katowice
source link:
https://liquipedia.net/starcraft2/IEM_Season_XIII_-_Katowice,
download link:
Not available at the time of publication.
- 2019 WCS Fall
source link:
https://liquipedia.net/starcraft2/2019_WCS_Fall,
download link:
https://drive.google.com/drive/folders/1LCt6xDRoLCHBaGo6Y-Be_K_bVCMedRSs.
- 2019 WCS Grand Finals
source link:
https://liquipedia.net/starcraft2/2019_WCS_Global_Finals,
download link:
https://bnetcmsus-a.akamaihd.net/cms/gallery/xg/XG44SSN4GZKW1572888219002.zip.
- 2019 WCS Spring
source link:
https://liquipedia.net/starcraft2/2019_WCS_Spring,
download link:
https://drive.google.com/drive/u/0/folders/1rcc3Ey-9ysnV90Auj9aT-eTw4sXNxWAR.
- 2019 WCS Summer
source link:

https://liquipedia.net/starcraft2/2019_WCS_Summer,
download link:
https://drive.google.com/drive/folders/1RASkC8X6rt9xSvtsbm7LbrurvC-YB3yr.

- 2019 WCS Winter
source link:
https://liquipedia.net/starcraft2/2019_WCS_Winter/Europe,
download link:
https://bnetcmsus-a.akamaihd.net/cms/page_media/6w/6WRTUZW0CNFV1554744096130.zip.
- 2020 Dreamhack Last Chance
source link:
https://liquipedia.net/starcraft2/ESL_Pro_Tour/2020/21/Masters/Last_Chance,
download link:
Not available at the time of publication.
- 2020 ASUS ROG Online
source link:
https://liquipedia.net/starcraft2/Assembly_Online/2020,
download link:
https://drive.google.com/uc?export=download&id=1TGZXNFfaUEzTqdbj36TEtanidgF-SzXV.
- 2020 Dreamhack SC2 Masters Fall
source link:
https://liquipedia.net/starcraft2/ESL_Pro_Tour/2020/21/Masters/Fall,
download link:
Not available at the time of publication.
- 2020 Dreamhack SC2 Masters Summer
source link:
https://liquipedia.net/starcraft2/ESL_Pro_Tour/2020/21/Masters/Summer,
download link:
Not available at the time of publication.
- 2020 Dreamhack SC2 Masters Winter
source link:
https://liquipedia.net/starcraft2/ESL_Pro_Tour/2020/21/Masters/Winter,
download link:
Not available at the time of publication.
- 2020 IEM Katowice
source link:
https://liquipedia.net/starcraft2/IEM_Katowice/2020,
download link:
https://drive.google.com/file/d/17aQ4YZySfcI84cxho5qmio2hB7QmEwRj/view.
- 2020 StayAtHome Story Cup 1
source link:
https://twitter.com/TrAiDoS/status/1249416661465210881?s=20,
download link:
https://drive.google.com/drive/folders/19t7UK9SjZniddkqSClsRxtEDUb-_T23E.
- 2020 StayAtHome Story Cup 2
source link:
https://liquipedia.net/starcraft2/StayAtHome_Story_Cup/2,
download link:
https://drive.google.com/drive/folders/1HHGmEmuxsI8ZLlxV7MQfRFaAX3bSGcD1.
- 2020 TSL5
source link:
https://liquipedia.net/starcraft2/TeamLiquid_StarLeague/5,
download link:
https://tl.net/downloads/TSL%205%20Replay%20Pack.zip.
- 2020 TSL6
source link:

https://tl.net/forum/starcraft-2/567627-tsl-6-replay-pack,
download link:
https://tl.net/downloads/TSL6%20Replay%20Pack.zip.

- 2021 03 Dreamhack SC2 Masters Fall
source link:
https://liquipedia.net/starcraft2/ESL_Pro_Tour/2021/22/Masters/Fall,
download link:
https://drive.google.com/file/d/12oEtS0tzMhSFZ3GYKV53z9v57jmF0Fay/view.
- 2021 ASUS ROG Fall
source link:
https://liquipedia.net/starcraft2/ASUS_ROG/2021/Fall,
download link:
https://drive.google.com/file/d/1GRN6VrKAV5JsEDUeVO_UhpDpZlibRFKl/edit.
- 2021 Cheeseadelphia Winter Championship
source link:
https://liquipedia.net/starcraft2/Cheeseadelphia/2021/Winter_Championship,
download link:
Not available at the time of publication.
- 2021 Dreamhack SC2 Masters Fall
source link:
https://liquipedia.net/starcraft2/ESL_Pro_Tour/2021/22/Masters/Fall,
download link: https://drive.google.com/file/d/12oEtS0tzMhSFZ3GYKV53z9v57jmF0Fay/view.
- 2021 Dreamhack SC2 Masters Summer
source link: https://liquipedia.net/starcraft2/ESL_Pro_Tour/2021/22/Masters/Summer,
download link:
Not available at the time of publication.
- 2021 Dreamhack SC2 Masters Winter
source link:
https://liquipedia.net/starcraft2/ESL_Pro_Tour/2021/22/Masters/Winter,
download link:
https://drive.google.com/file/d/16ZV2x7BBfSoYWed2jsyLdsA566ROmKSn/view.
- 2021 IEM Katowice
source link:
https://liquipedia.net/starcraft2/IEM_Katowice/2021,
download link:
Not available at the time of publication.
- 2021 StayAtHome Story Cup 3
source link:
https://twitter.com/TrAiDoS/status/1371219576407654405,
download link:
https://drive.google.com/drive/folders/1fF0Cn0TrMHKLIL8fVPypWoJzwU2EZduv.
- 2021 StayAtHome Story Cup 4
source link:
https://liquipedia.net/starcraft2/StayAtHome_Story_Cup/4,
download link:
https://drive.google.com/drive/folders/1nKCZOagxHMgUpV7BefmjRVN0Ib-qoOs3.
- 2021 TSL7
source link:
https://liquipedia.net/starcraft2/TeamLiquid_StarLeague/7,
download link:
https://drive.google.com/file/d/14b-J6ijTEHH0daI-9sHKDIa-OggetlWC/view.
- 2021 TSL8
source link:
https://liquipedia.net/starcraft2/TeamLiquid_StarLeague/8,
download link:
https://drive.google.com/file/d/11QX1g9XOSgWIC1IAmyeeBASNmuC8-vWo/view.

- 2022 Dreamhack SC2 Masters Last Chance 2021
  source link:
  https://liquipedia.net/starcraft2/ESL_Pro_Tour/2021/22/Masters/Last_Chance,
  download link:
  Not available at the time of publication.

- 2022 IEM Katowice
  source link:
  https://liquipedia.net/starcraft2/IEM_Katowice/2022,
  download link:
  https://drive.google.com/file/d/1__YogjKb4OTUJroJ-J1YVNUzVhyRZFw6/view.

2 Additional Figures

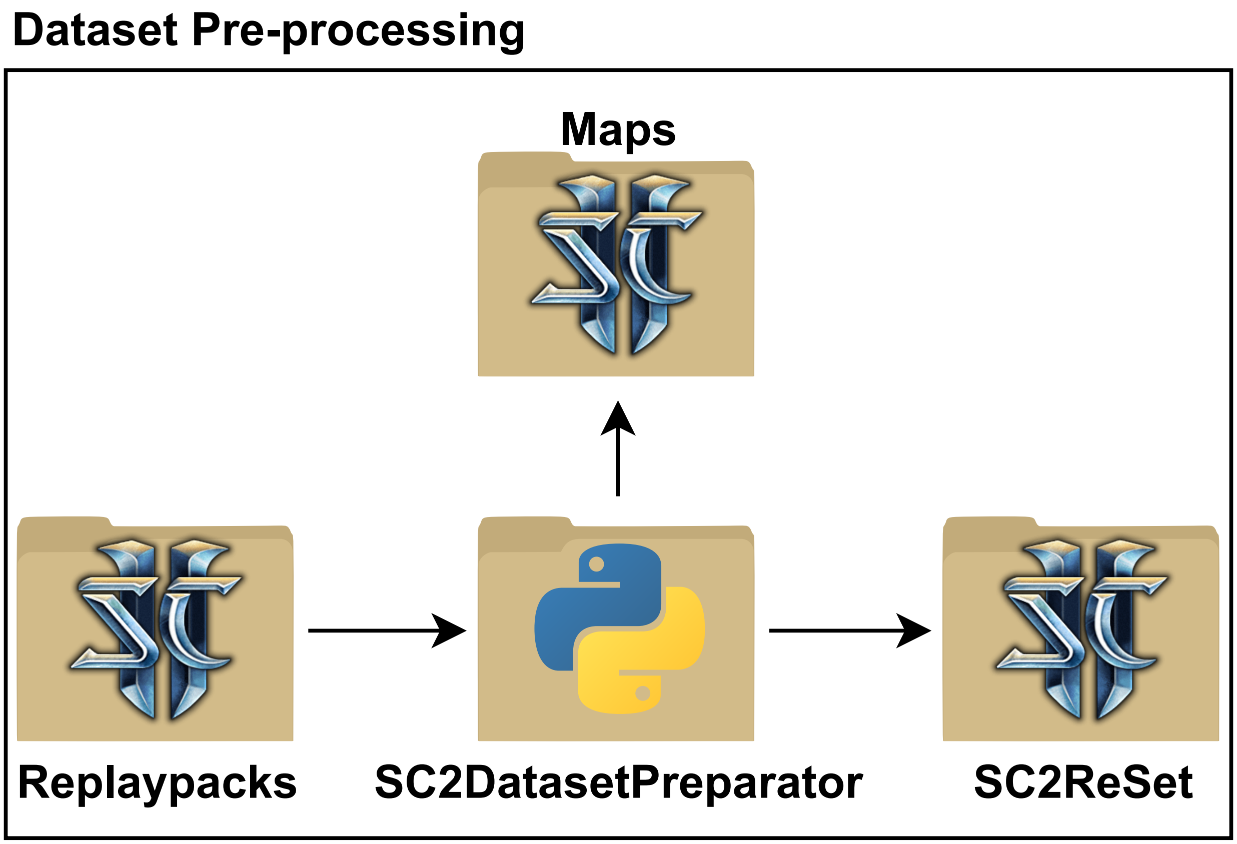

Figure 1: Pre-processing used to create SC2ReSet at the time of publication [1].

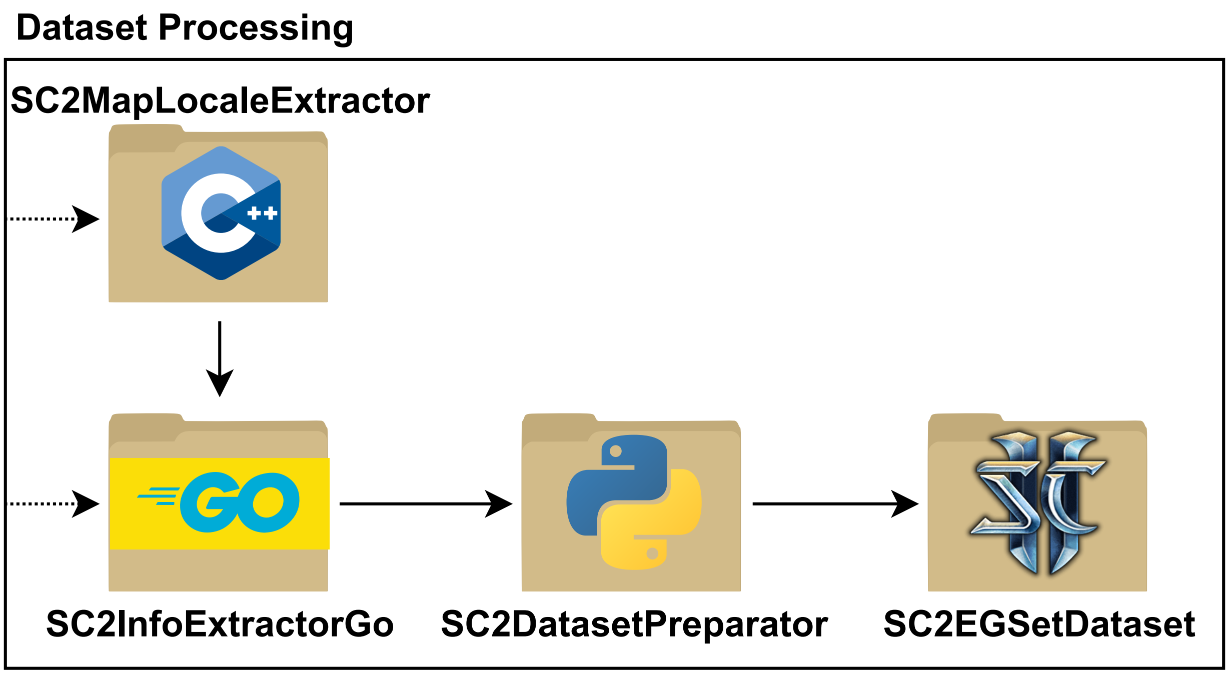

Figure 2: Processing pipeline used to create SC2EGSetDataset [2]. Including tools such as SC2DatasetPreparator [3], SC2MapLocaleExtractor [4], and SC2InfoExtractorGo [5].

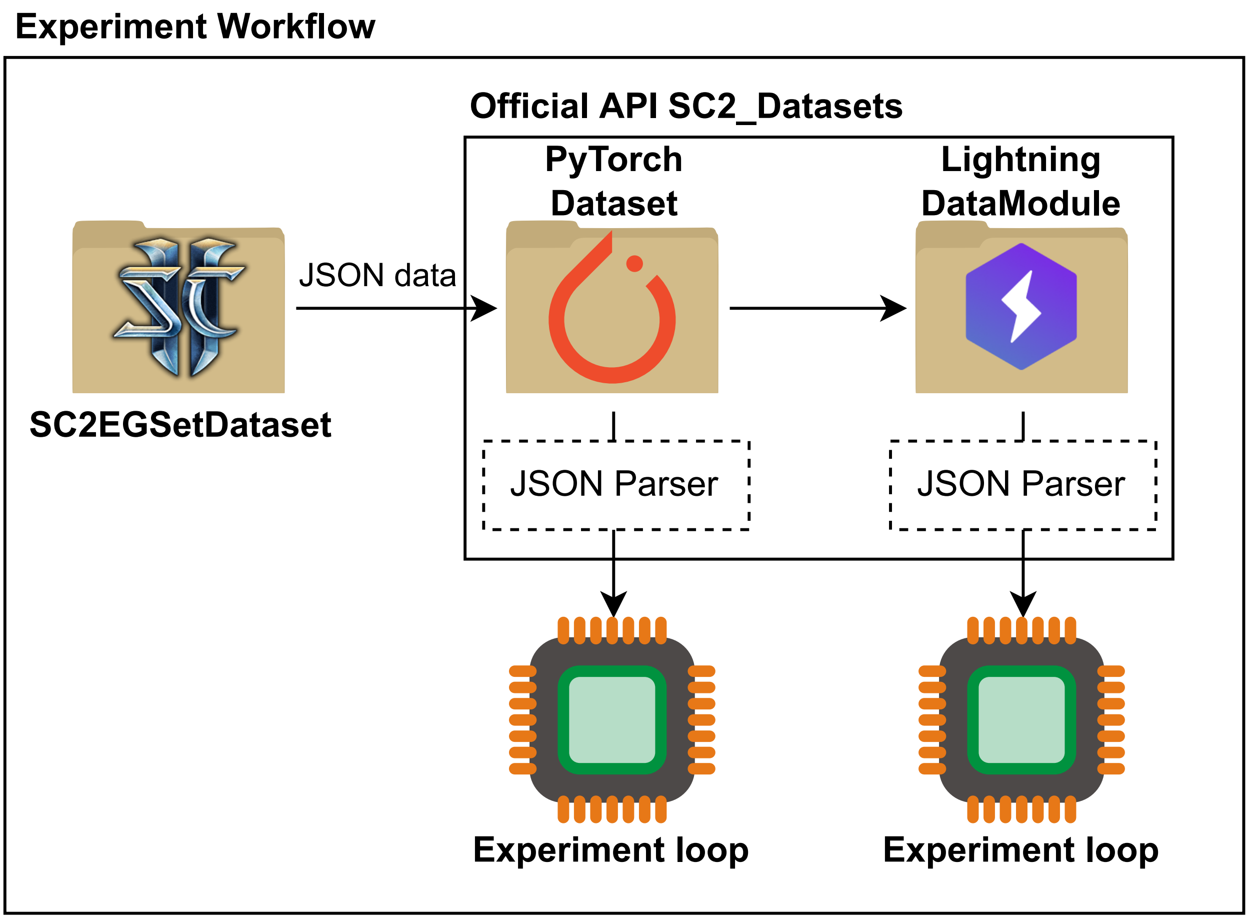

Figure 3: Visual example of using the SC2EGSetDataset [2] with the officially provided API [6] to conduct experiments.

References

[1] A. Białecki, "SC2ReSet: StarCraft II Esport Replaypack Set," *Zenodo*, jun 2022. [Online]. Available: https://doi.org/10.5281/zenodo.5575796 1, 6

[2] A. Białecki, N. Jakubowska, P. Dobrowolski, P. Białecki, L. Krupiński, A. Szczap, R. Białecki, and J. Gajewski, "SC2EGSet: StarCraft II Esport Game State Dataset," *Zenodo*, jun 2023. [Online]. Available: https://doi.org/10.5281/zenodo.5503997 7, 8

[3] A. Białecki, P. Białecki, and L. Krupiński, "Kaszanas/SC2DatasetPreparator: 1.2.0 SC2DatasetPreparator Release," jun 2022. [Online]. Available: https://doi.org/10.5281/zenodo.5296664 7

[4] A. Białecki and P. Białecki, "Kaszanas/SC2MapLocaleExtractor: 1.1.1 SC2MapLocaleExtractor Release," aug 2021. [Online]. Available: https://doi.org/10.5281/zenodo.4733264 7

[5] A. Białecki, L. Krupiński, and P. Białecki, "Kaszanas/SC2InfoExtractorGo: 1.2.1 SC2InfoExtractorGo Release," jun 2022. [Online]. Available: https://doi.org/10.5281/zenodo.5296788 7

[6] A. Białecki, P. Białecki, A. Szczap, and L. Krupiński, "Kaszanas/SC2_Datasets: 1.0.0 SC2_Datasets Release," jul 2022. [Online]. Available: https://doi.org/10.5281/zenodo.6629005 8
